# Schizophrenia Hypothesis: Autonomic Nervous System Dysregulation of Fetal and Adult Immune Tolerance

**DOI:** 10.3389/fnsys.2022.844383

**Published:** 2022-07-01

**Authors:** Tamara Carnac

**Affiliations:** Independent Researcher, Northampton, United Kingdom

**Keywords:** schizophrenia, immune, Akt, cytokines, gut, GABA, norepinephrine, autonomic nervous system

## Abstract

The autonomic nervous system can control immune cell activation via both sympathetic adrenergic and parasympathetic cholinergic nerve release of norepinephrine and acetylcholine. The hypothesis put forward in this paper suggests that autonomic nervous system dysfunction leads to dysregulation of immune tolerance mechanisms in brain-resident and peripheral immune cells leading to excessive production of pro-inflammatory cytokines such as Tumor Necrosis Factor alpha (TNF-α). Inactivation of Glycogen Synthase Kinase-3β (GSK3β) is a process that takes place in macrophages and microglia when a toll-like receptor 4 (TLR4) ligand binds to the TLR4 receptor. When Damage-Associated Molecular Patterns (DAMPS) and Pathogen-Associated Molecular Patterns (PAMPS) bind to TLR4s, the phosphatidylinositol-3-kinase (PI3K)-protein kinase B (Akt) pathway should be activated, leading to inactivation of GSK3β. This switches the macrophage from producing pro-inflammatory cytokines to anti-inflammatory cytokines. Acetylcholine activation of the α7 subunit of the nicotinic acetylcholine receptor (α7 nAChR) on the cell surface of immune cells leads to PI3K/Akt pathway activation and can control immune cell polarization. Dysregulation of this pathway due to dysfunction of the prenatal autonomic nervous system could lead to impaired fetal immune tolerance mechanisms and a greater vulnerability to Maternal Immune Activation (MIA) resulting in neurodevelopmental abnormalities. It could also lead to the adult schizophrenia patient’s immune system being more vulnerable to chronic stress-induced DAMP release. If a schizophrenia patient experiences chronic stress, an increased production of pro-inflammatory cytokines such as TNF-α could cause significant damage. TNF-α could increase the permeability of the intestinal and blood brain barrier, resulting in lipopolysaccharide (LPS) and TNF-α translocation to the brain and consequent increases in glutamate release. MIA has been found to reduce Glutamic Acid Decarboxylase mRNA expression, resulting in reduced Gamma-aminobutyric acid (GABA) synthesis, which combined with an increase of glutamate release could result in an imbalance of glutamate and GABA neurotransmitters. Schizophrenia could be a “two-hit” illness comprised of a genetic “hit” of autonomic nervous system dysfunction and an environmental hit of MIA. This combination of factors could lead to neurotransmitter imbalance and the development of psychotic symptoms.

## Introduction

Schizophrenia is a debilitating mental illness which has a global prevalence of approximately 0.28% (Charlson et al., 2018). Although antipsychotic medications exist to treat schizophrenia, these medications are not effective at treating all patients and they often have side effects (Muench and Hamer, 2010). Therefore there is an unmet clinical need for more effective treatments to be developed for this condition.

Schizophrenia is characterized by heterogeneous positive and negative symptom groups. Negative symptoms describe a reduction or absence of normal healthy behaviors and are grouped into five main constructs: alogia (a reduction in the amount of words spoken), asociality, blunted affect, avolition (reduced motivation and desire to achieve goals), and anhedonia (reduced ability to experience pleasure). Up to 60% of patients may experience clinically relevant negative symptoms, and a considerable proportion of patients do not see an improvement of these symptoms in response to antipsychotic treatment.

Positive symptoms describe a distortion or excess of normal function including symptoms such as hallucinations, disorganized behavior, delusions and thought disorder. Approximately 30% of schizophrenia patients are considered to be treatment-resistant, meaning that they don’t respond to two trials of dopaminergic antipsychotics.

In recent years, much research has focused on the immune system in schizophrenia and there is a growing consensus for a key involvement of the immune system in schizophrenia (van Kesteren et al., 2017; Pollak et al., 2020). It is widely believed that schizophrenia is caused by a combination of genetic and environmental factors. Much research has focused on the concept of schizophrenia being a “two-hit” illness, with a prenatal first hit that makes a person more vulnerable to a later second hit (Bayer et al., 1999; Feigenson et al., 2014).

In the theory being proposed in this paper, it is suggested that schizophrenia is a “two-hit” illness of environmental and genetic factors that result in a genetic dysregulation of the sympathetic and/or parasympathetic nervous system, leading to impaired mechanisms of fetal immune tolerance that lead to abnormal prenatal neurodevelopment in response to maternal immune activation. These factors could culminate in an imbalance of neurotransmitters, with impaired inhibitory GABAergic signaling leading to overexcitable and unsynchronized neuronal firing (Petty, 1995; Hamm et al., 2017).

Gaining a better understanding of the pathophysiology of schizophrenia may help researchers to develop more effective and customized treatments for patients.

### Peripheral Immune Activation in Schizophrenia Patients

There are some indications of the existence of peripheral immune activation in schizophrenia patients. Recent research has shown that peripheral blood serum from first-episode drug-naive schizophrenia patients can provoke microglial pro-inflammatory activation (van Rees et al., 2018). What this could indicate is the presence of high levels of immune cell activators like Pathogen-Associated Molecular Pattern (PAMPS) or Damage-Associated Molecular Patterns (DAMPS) in the blood serum of schizophrenia patients and it is possible that these circulating immune activators could activate peripheral immune cells, and pass through the blood brain barrier to activate microglia.

Rat/mouse models are useful tools to try to understand the effect of schizophrenia risk genes on the peripheral immune system. Using a rat model to over-express the schizophrenia risk gene DISC1, researchers discovered a number of dysregulated genes in the rats’ peripheral blood mononuclear cells (Trossbach et al., 2019).

Peripheral regulatory T (Treg) cell activation has also been found to be reduced in schizophrenia patients and also increased by antipsychotic treatment, with higher levels of Treg cells correlating with fewer negative symptoms after antipsychotic treatment (Kelly et al., 2018; Sahbaz et al., 2020).

An interesting connection between possible immune system dysregulation and the metabolic dysregulation that is frequently observed in schizophrenia patients was discovered by researchers when they observed that schizophrenia patient peripheral monocytes have alterations in insulin receptor expression. This could potentially indicate that in some patients, insulin signaling may be involved in peripheral immune dysregulation (Lago et al., 2021).

It has also been found that higher levels of Th17 cells have been found in the plasma of schizophrenia patients, with Th17 levels significantly reducing after 4 weeks of risperidone treatment (Ding et al., 2014).

IL-23 is released from activated macrophages and dendritic cells and has been found to be increased in the blood serum of schizophrenia patients. This indicates a possible dysregulation of macrophages and/or dendritic cells (Borovcanin et al., 2015). Increased IL-23 levels can lead to increased Th17 cell activation, which could explain the higher levels of Th17 cells found in schizophrenia patients (Maloy and Kullberg, 2008).

There is a dearth of research into the function of immune cells of the intestinal wall in schizophrenia patients. Conventional dendritic cells and macrophages are found throughout the intestinal immune system, but much remains to be understood about the function of each cell type. In part, this is due to the fact that intestinal immune cells are difficult to isolate, but also the idea that the gut might have an involvement in the pathophysiology of schizophrenia is still a relatively new concept in schizophrenia research. Circulating monocytes can infiltrate inflamed or mucosal tissues where they differentiate into either dendritic cells or macrophages. Unlike resident macrophages in other tissues, macrophages in the intestines are constantly replenished from circulating monocytes that differentiate in the mucosa (Bain and Mowat, 2014).

It is therefore a possibility that the dysregulation found in the circulating peripheral monocytes of schizophrenia patients could also be present in the immune cells of the intestinal wall, although further research of the intestinal immune system in schizophrenia patients is needed to investigate this concept.

### Mechanisms of Immune Cell Tolerance

With a number of studies indicating that there may be peripheral immune cell dysregulation in schizophrenia patients, the possibility of there being dysregulated mechanisms of immune cell tolerance is quite plausible.

Endotoxin tolerance is a property of macrophages and microglia that leads to a different response to repetitive or chronic exposure to bacterial endotoxins (Seeley and Ghosh, 2017). At first, when exposed to gram-negative bacterial endotoxin lipopolysaccharide (LPS), there is a strong inflammatory macrophage reaction with a release of pro-inflammatory cytokines. In normal functioning macrophages, this release of pro-inflammatory cytokines should be followed by a release of anti-inflammatory cytokines, with macrophages being more tolerant to LPS challenge in the future, releasing less pro-inflammatory cytokines. Immune tolerance is an important part of a healthy and functional immune system.

Toll-like receptor 4 (TLR4) is a transmembrane protein which belongs to the pattern recognition receptor family. TLR4 has long been recognized as the sensing receptor for PAMPS such as LPS. In addition, it also binds molecules produced as a result of tissue injury such as DAMPs (Kawasaki and Kawai, 2014). TLR4s are found on the surface of macrophages, microglia and neurons. When LPS and DAMPs bind to TLR4, a signaling cascade is triggered which leads to the production of cytokines.

The production of these cytokines is partly mediated by TLR4 activation of the phosphatidylinositol-3-kinase-protein kinase B (PI3K/Akt) pathway and phosphorylation of Glycogen Synthase Kinase-3β (GSK3β). In basal conditions GSK3β is constitutively active, and requires phosphorylation to inactivate it. As well as having it’s own circadian rhythmicity, which influences it’s phosphorylation, GSK3β is also negatively regulated via phosphorylation by Akt. Research has found a reduction of AKT1-associated pathways in the peripheral blood mononuclear cells of schizophrenia patients (van Beveren et al., 2012).

GSK3β inhibits the binding of cAMP-response element binding protein (CREB) to CREB-binding protein (CBP) (Grimes and Hope, 2001), whereas, it promotes the binding of Nuclear factor kappa B (NF-kB) to CBP, leading to reduced anti-inflammatory cytokine production and enhanced pro-inflammatory production from immune cells, respectively (Cortés-Vieyra et al., 2012). Taking into account it’s circadian rhythmicity, if GSK3β is active when TLR4s are initially activated, in a healthy person there should be an immediate release of pro-inflammatory cytokines, however LPS stimulation of TLR4s should also activate the PI3K/Akt pathway, which would normally result in phosphorylation and inactivation of GSK3β. This would result in a switch from production of pro-inflammatory cytokines to anti-inflammatory cytokines in macrophages and microglia (Beurel et al., 2010; Ko and Lee, 2016).

If a dysregulated PI3K/Akt pathway in the immune cells of schizophrenia patients is preventing the immune tolerance mechanism from functioning correctly, macrophages could be more likely to have an excessive or prolonged inflammatory reaction to DAMPs and PAMPs such as LPS. DAMPs are released from damaged or dying cells (Vénéreau et al., 2015). High mobility group box-1 (HMGB-1) is an example of a DAMP and research has found it is released in response to social defeat stress (Velazquez et al., 2017; Zhang et al., 2019). Research has found that a psychotic episode is often triggered by a period of stress in a schizophrenia patient’s life (Selten et al., 2013).

**FIGURE 1:**
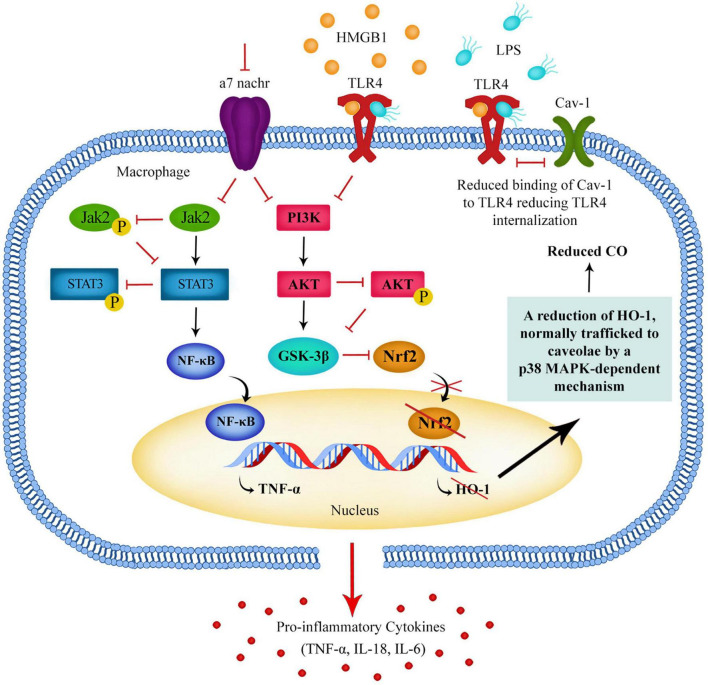
Downregulation of the Jak2/STAT3 pathway and PI3K/Akt pathway in macrophages leads to a reduction of GSK3β phosphorylation resulting in GSK3β remaining in an active state. Active GSK3β promotes the activation of NF-kB and the production of pro-inflammatory cytokines. These pathways can be regulated by α7 nAChR activation and α7 nAChRs can regulate TLR4 surface expression, possibly via a Nrf2-HO-1-CO-TLR4 pathway.

In a healthy person, a release of pro-inflammatory cytokines should help to destroy a damaged cell or fight pathogens and bacteria, followed by a switch to anti-inflammatory cytokines to compensate for unwanted damage the pro-inflammatory cytokines might have caused (Cortés-Vieyra et al., 2021). If the PI3K/Akt pathway is dysregulated in schizophrenia patients, when DAMPs and LPS act upon TLR4s, the PI3K/Akt pathway may not be activated in the way it normally would be, and this could mean that GSK3β remains active in schizophrenia patients, resulting in a prolonged or exaggerated production of pro-inflammatory cytokines (Figure 1).

Modulation of the PI3K/Akt signaling pathway can tip the balance of the immune response between tolerance and inflammation. It could be possible that if the PI3K/Akt pathway is dysregulated in schizophrenia patients, their immune response could be altered. The presence of abnormally high levels of pro-inflammatory cytokines such as Tumor Necrosis Factor alpha (TNF-α) and Interleukin 6 (IL-6) found in schizophrenia patients indicates that in some patients it is possible that the immune system balance is being tipped toward a more inflammatory state (Lee et al., 2016).

As well as inherent genetic dysregulation of the immune cell affecting the regulation of the PI3K/Akt pathway and immune tolerance mechanisms, it should also be considered that there could be dysregulation of the circadian rhythmicity of GSK3β phosphorylation. Another factor to consider is that the PI3K/Akt pathway of the immune cell can be controlled by extrinsic mechanisms via receptors present on the cell surface of immune cells.

### α7 Nicotinic Acetylcholine Receptor Control of the PI3K/Akt Pathway

Although there are many types of receptors present on immune cells, the α7 subunit of the nicotinic acetylcholine receptor (α7 nAChR) in particular has been shown to control pro-inflammatory cytokine release (Mashimo et al., 2019). Research shows that α7 nAChR activation on macrophages reduces the production of pro-inflammatory cytokines, including TNF-α (Parrish et al., 2008). Research also indicates that the α7 nAChR can reduce the expression of TLR4 expression via the PI3K/AKT pathway, which could be part of the mechanism of developing endotoxin tolerance, as a reduction of TLR4 expression on the cell surface may limit the immune cell response to LPS (Hamano et al., 2006; Tyagi et al., 2010).

In a healthy person, when LPS acts upon TLR4s on immune cells, the PI3K/Akt pathway should be activated (Vergadi et al., 2017). The PI3K/Akt pathway plays a crucial role in modulating Nuclear factor-erythroid factor 2-related factor 2 (Nrf2) protein expression in macrophages (Xu et al., 2015). TLR4 agonist binding should result in Nrf2 nuclear translocation via the PI3K/Akt pathway, leading to increased expression of antioxidant enzyme heme oxygenase-1 (HO-1). Research has shown that this results in increased levels of carbon monoxide (CO), generated by HO-1. It has been found that CO suppresses membrane activation of TLR4 by blocking TLR4 glycosylation and the physical interaction between myeloid differentiation factor-2 (MD-2), caveolin-1 and TLR4 (Wang et al., 2009; Riquelme et al., 2015).

This possible PI3K-Akt-Nrf2-HO-1-CO-TLR4 pathway could explain part of the process by which macrophages reduce TLR4 expression when developing tolerance to LPS and HMGB-1. It may also be a crucial pathway activated by acetylcholine (ACh) binding of α7 nAChRs, and explain how activation of α7 nAChR leads to reduced TLR4 expression (Figure 1).

If schizophrenia patients have reduced ACh release due to dysfunction of the sympathetic or parasympathetic nervous system, this pathway could be down-regulated and lead to increased membrane expression of TLR4. This may in some way explain the enhanced peripheral TLR responses found in psychosis patients (McKernan et al., 2011). Research has shown that not only do antipsychotic-naive schizophrenia patients have an increased percentage of TLR4+ monocytes compared to healthy controls, but the percentage of TLR4+ monocytes decreases after antipsychotic treatment (Kéri et al., 2017).

A possible down-regulation of the PI3K-Akt-Nrf2-HO-1-CO-TLR4 pathway is given credibility by research that shows down-regulation of the components of this pathway in schizophrenia patients. As well as the down-regulated Akt-related pathways that have been found in peripheral mononuclear cells of schizophrenia patients, there is also evidence of reduced Nrf2 and HO-1 gene expression in peripheral lymphocytes of acute psychosis patients (Shmarina et al., 2020).

A deficiency of α7 nAChR expression has consistently been found in schizophrenia patients, as well as an association of the cholinergic nicotinic receptor gene alpha 7 subunit (CHRNA7) (Perl et al., 2003; Bakanidze et al., 2013; Young and Geyer, 2013). There have also been promising trials which have shown the efficacy of α7 nAChR agonists for cognitive deficits in schizophrenia patients (Olincy et al., 2006).

As well as regulating the PI3K/Akt pathway, α7 nAChRs have also been shown to regulate pro-inflammatory cytokine release via activation of the Jak2/STAT3 signaling pathway. When Jak2 phosphorylates STAT3, it binds to the p65 subunit of NF-κB and blocks the transcription of NF-κB, which prevents pro-inflammatory cytokine release (Yang et al., 2015; Tan et al., 2022). Research into peripheral lymphocyte signaling in antipsychotic-naive schizophrenia patients found not only reduced Akt1 levels but also that the level of Stat3 phosphorylation in immune cells predicted improvements in general psychopathology scores (Lago et al., 2022).

### Stress-Induced Immune Activation

In a healthy person experiencing a period of stress, DAMPs may be released by cells and activate TLR4s and the receptor for advanced glycation end product (RAGE) on macrophages, dendritic cells and microglia. It is likely that in a healthy person, macrophages and microglia should develop some level of immune tolerance to stress molecules such as HMGB-1 (Watanabe and Son, 2021). This means that if a healthy person experiences months or years of stress, their immune cells should become desensitized to stress molecules and not have a prolonged pro-inflammatory response.

If schizophrenia patients have a problem with developing immune tolerance, this could make them more sensitive to stress. Rather than reducing their immune response to DAMPs over time, their immune system may have a prolonged inflammatory activation by DAMPs such as HMGB1 and therefore release pro-inflammatory cytokines in response to chronic stress (Figure 1). Inflammatory cytokines and LPS also upregulate HMGB1 expression and secretion from macrophages, further increasing circulating HMGB1 (Chen et al., 2022). This may not cause much damage during a short stressful experience, but if a patient is producing pro-inflammatory cytokines such as TNF-α over a long period of chronic stress lasting months or years, it is possible that significant damage could be caused.

### Vagus Nerve Regulation of the Inflammatory Immune Response

**FIGURE 2:**
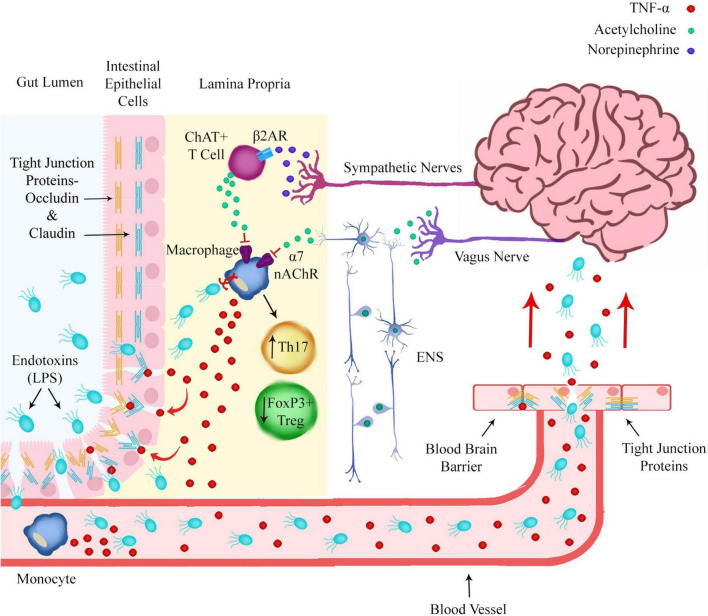
Norepinephrine and acetylcholine release from sympathetic nerves and the enteric nervous system (ENS) in the intestinal wall regulates pro-inflammatory polarization of immune cells. Prolonged macrophage production of pro-inflammatory cytokines such as TNF-α could lead to degradation of the intestinal epithelial barrier and blood brain barrier by modulating the expression of tight junction proteins. This would allow endotoxins such as LPS to translocate from the gut lumen to the brain via the circulatory system.

Immune cells of the intestinal wall are regulated by both sympathetic and parasympathetic innervation (Figure 2). The vagus nerve is the main nerve of the parasympathetic nervous system and regulates many functions of the body including the innate immune response.

The cholinergic neurons of the vagus nerve innervate the celiac ganglion, from which originates the sympathetic splenic nerve. Via this pathway the vagus nerve can inhibit the immune response of immune cells in the spleen and inhibit TNF-α production (Rosas-Ballina et al., 2008). The vagus nerve can also inhibit the inflammatory response of immune cells resident in the intestinal wall via the enteric nervous system (ENS) (de Jonge, 2013).

Stimulation of the vagus nerve leads to the release of ACh, the neurotransmitter which binds to the α7 nAChR. The α7 nAChR is expressed on the cell surface of macrophages and other cytokine producing immune cells and ACh binding to α7 nAChR inhibits the release of pro-inflammatory cytokines. This process is referred to as the cholinergic anti-inflammatory pathway (Goverse et al., 2016).

It has previously been suggested that the vagus nerve and α7 nAChRs may be involved in the pathophysiology of schizophrenia (das Graças Corsi-Zuelli et al., 2017). While it is possible that alteration of gene expression in the immune cell itself causes dysregulation of the PI3K/Akt pathway, it is also possible that reduced activation of the α7 nAChR on the immune cell surface leads to downregulation of the PI3K/Akt pathway in immune cells.

### Sympathetic Nerve Regulation of the Inflammatory Immune Response

Sympathetic nerves have also been shown to regulate the inflammatory state of immune cells, either through a direct pathway of sympathetic nerves, or as part of the vagus nerve pathway in concert with cholinergic nerves. It is therefore possible that in some schizophrenia patients, it could be dysregulation of the adrenergic nerves of the sympathetic nervous system that results in an inability to trigger release of ACh from ACh-producing T cells (ChAT+ T cells) in the spleen and intestinal wall. ChAT+ T cells are T-cells that express the enzyme choline acetyltransferase (ChAT) and produce ACh (Brinkman et al., 2019).

Sympathetic adrenergic nerves in the spleen and intestinal wall release norepinephrine (NE), which binds to the beta-2 adrenergic receptor (β2AR) on ChAT+ T cells, triggering the release of ACh (Figure 2). Inhibition of NE release from sympathetic nerves or reduced expression or sensitivity of β2ARs on adrenergic neurons or ChAT+ T cells could lead to reduced ACh release, reduced activation of α7 nAChR on macrophages and an inability to inhibit the pro-inflammatory production of cytokines by macrophages and dendritic cells.

It is possible that dysfunction of the adrenergic nerves of the sympathetic nervous system of schizophrenia patients could lead to defective immune tolerance mechanisms either via reducing NE action upon immune cell β2ARs or by modulating ACh action on immune cell α7 nAChRs.

### Presynaptic Norepinephrine Transporters

There are some indications that dysfunctional adrenergic neuronal control of NE release or clearance could be occurring in some schizophrenia patients. Many types of antipsychotics have been found to increase NE levels considerably, and the atypical antipsychotic clozapine acts as an antagonist at alpha(2C) adrenergic receptors (Kalkman and Loetscher, 2003; Boyda et al., 2020).

Clozapine has also been shown to influence the expression of the norepinephrine transporter (NET), which is responsible for the rapid clearance of NE (Yoshimura et al., 2000). Presynaptic alpha 2 adrenergic receptors are supposed to act as part of a negative feedback mechanism to reduce NE release in response to extracellular NE levels, so it is possible that clozapine could be interfering with this negative feedback mechanism in order to increase extracellular NE levels (Philipp et al., 2002).

It is possible that in schizophrenia patients, genetic dysregulation of pathways controlling NET expression could prevent the regulation of extracellular NE levels, leading to dysregulation of the adrenergic nerves of the sympathetic nervous system.

### Increased Norepinephrine Transporter Expression

Many studies have found there to be reduced dopamine (DA) release in the prefrontal cortex in schizophrenia patients (Slifstein et al., 2015). In the prefrontal cortex both NE and DA are transported out of the extracellular space into the presynaptic adrenergic neuron by NET. While DA uptake in the nucleus accumbens and the caudate depends primarily on dopamine transporter (DAT), DA uptake in the prefrontal cortex depends primarily on NET (Morón et al., 2002).

Researchers at Vanderbilt University Medical Centre published research in 2010 that suggests there could be a link between NET expression and the hypodopaminergia found in the prefrontal cortex of schizophrenia patients. The researchers were attempting to see if there is a mechanism linking low DA in the prefrontal cortex with aberrant Akt signaling, as reduced Akt phosphorylation at Ser473 has been observed in postmortem brains and lymphocytes of schizophrenia patients (Siuta et al., 2010).

**FIGURE 3:**
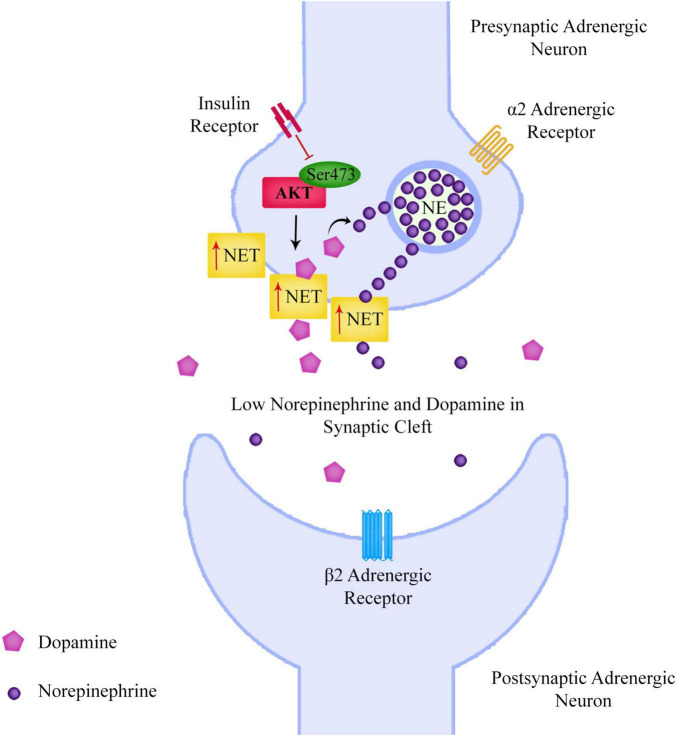
Reduced neuronal Akt Ser473 phosphorylation leads to increased surface norepinephrine transporter expression, leading to an increased neuronal uptake of both norepinephrine and dopamine from the extracellular space, resulting in increased presynaptic conversion of dopamine to norepinephrine, with consequent lower levels of dopamine and higher levels of norepinephrine in vesicles of the presynaptic neurons. This could lead to lower levels of dopamine in the prefrontal cortex and also affect the signaling of the sympathetic nervous system.

The researchers eliminated the mTORC2 regulatory protein rictor in the neurons of mice, leading to impairments in Akt Ser473 phosphorylation. The mice not only showed deficits in prepulse inhibition (PPI) similar to schizophrenia patients, they showed increased NET expression, reduced DA and increased cortical NE levels. The researchers proposed that the increased NET expression resulted in increased neuronal uptake of both NE and DA from the extracellular space, resulting in increased presynaptic conversion of DA to NE, with consequent lower levels of DA and higher levels of NE in vesicles of the presynaptic neurons. The researchers gave further strength to their hypothesis, when they discovered blocking NET in these mice corrected PPI and increased cortical DA levels (Figure 3).

### Norepinephrine Transporter Expression in the Sympathetic Nervous System

As the sympathetic nervous system comprises of adrenergic nerves expressing NET, it is possible that impaired Akt phosphorylation in adrenergic nerves could lead to changes in NET expression that could dysregulate the sympathetic nervous system. There are some indications that the sympathetic nervous system is dysregulated in schizophrenia patients and that this could be due to increased NET expression.

The sympathetic nervous system regulates cardiac function and can affect heart rate and the output of the heart by releasing NE. Research has consistently shown lower left ventricular mass in the hearts of schizophrenia patients (Pillinger et al., 2019). However research has also shown increased cardiac sympathetic activity is coupled to increased left ventricular mass (Burns et al., 2007). What this could indicate is that the reduced left ventricular mass found in schizophrenia patients could be due to dysregulated cardiac sympathetic activity. Left ventricular mass correlates with the level of NE release, and research has shown that NET binding corresponds to a high NE uptake in the ventricles. Interestingly, NET binding is significantly higher in the left ventricle compared to the right ventricle, which might explain why the left ventricle in particular has lower mass in schizophrenia patients (Wehrwein et al., 2008).

The sympathetic nervous system is also involved in control of the functions of the intestines. Schizophrenia patients have been shown to have an increased risk of colon cancer- estimated by one study to be a 190% increased risk (Hippisley-Cox et al., 2007). Research has also shown that NET is highly expressed in human colon cancer cells, and also that β2ARs are significantly associated with tumor size in colon cancer patients (Ciurea et al., 2017; Zhang et al., 2020).

As well as cardiac and intestinal regulation, sympathetic nerves are also involved in sleep regulation. The pineal gland receives sympathetic innervation from the superior cervical ganglion (SCG). The suprachiasmatic nucleus (SCN) is involved in the control of circadian rhythms and, via sympathetic innervation from the SCG, activates arylalkylamine *N*-acetyltransferase (AA-NAT) in the pineal gland, which is the main enzyme regulating melatonin synthesis (Lumsden et al., 2020).

While studies on melatonin levels have provided mixed results, there are indications that the melatonin circadian rhythm is dysregulated in schizophrenia patients (Anderson and Maes, 2012). It is possible that dysregulated NET expression on sympathetic nerves could reduce adrenergic transmission, preventing melatonin synthesis in the pineal gland. Interestingly, melatonin has also been shown to affect macrophage polarization, inhibiting M1 polarization and promoting M2 polarization, which means that low levels of circulating melatonin in schizophrenia patients could further increase the pro-inflammatory polarization of peripheral immune cells (Yi and Kim, 2017).

### Norepinephrine Release During Stress

**FIGURE 4:**
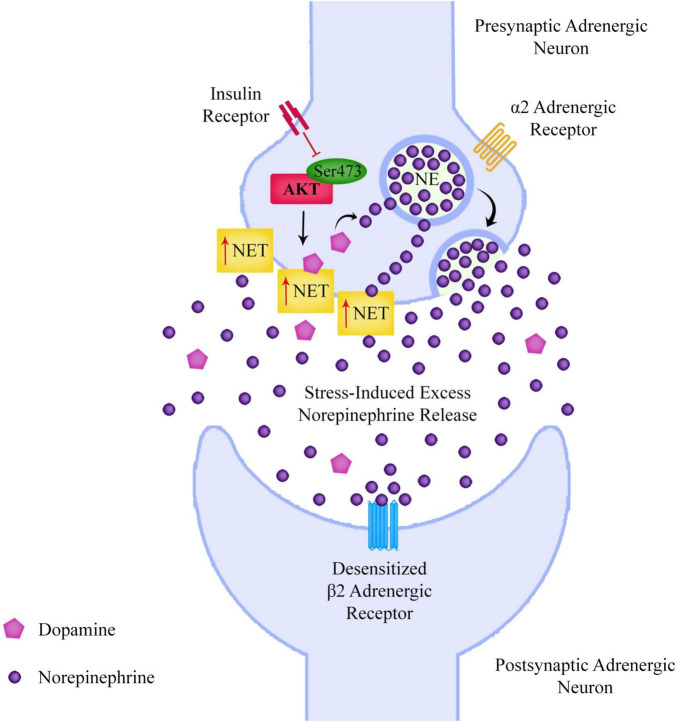
If reduced Akt Ser473 phosphorylation is leading to increased adrenergic surface norepinephrine transporter expression and a consequent build-up of presynaptic norepinephrine, this could lead to excessive norepinephrine release during periods of stress, which could desensitize adrenergic receptors such as the beta-2 adrenergic receptor.

If dysregulation of Akt phosphorylation at Ser473 is leading to increased presynaptic NET expression, this could lead to a build-up of NE in the presynaptic neuron. As NE is released during stress, this could mean that schizophrenia patients may have low levels of extracellular NE when they are not stressed, but could have a larger than average NE release during stress due to the increased presynaptic neuronal NE content (Figure 4). β2AR agonists including NE lead to desensitization of β2ARs, a mechanism that is displayed in the pineal gland, with β2ARs being supersensitive in the evening after a period of low NE during the day, and considerably less sensitive in the morning after being exposed to higher levels of NE overnight (Romero and Axelrod, 1974; Vatner et al., 1989).

If a schizophrenia patient experiences chronic stress over a long period of time, this could lead to high levels of NE release into the extracellular space, leading to chronic desensitization of β2ARs. Research has found that prolonged agonist exposure can lead to a reduction in β2AR mRNA (Danner et al., 1998). Although it has already been discussed that dysregulated immune tolerance mechanisms in immune cells could make schizophrenia patients more vulnerable to DAMP and PAMP activation, it is also possible that stress-induced desensitization of β2ARs could actually dysregulate the sympathetic nervous system and affect immune tolerance mechanisms.

As NE release from sympathetic neurons suppresses pro-inflammatory activation of immune cells in the spleen and intestinal wall, low levels of NE in the synaptic clefts of the sympathetic nervous system could lead to a lack of suppression of the inflammatory immune response. However it’s possible that this could be further worsened by a long period of stress leading to desensitization and mRNA downregulation of β2ARs.

The same effect could be happening to microglia in the Central Nervous System (CNS), which also express β2ARs, and reduce production of pro-inflammatory cytokines in response to NE (Sugama et al., 2019). This could play a part in contributing to the pro-inflammatory activation of microglia found in schizophrenia patients (van Berckel et al., 2008).

### Catechol-*O*-Methyl-Transferase and Norepinephrine Clearance

If β2AR mRNA expression can become down-regulated by prolonged agonist exposure, this effectively means that very low levels of NE in the synaptic cleft and very high levels could actually have the same effect.

Norepinephrine is metabolized by catechol-*O*-methyl-transferase (COMT) or monoamine oxidase (MAO), or deactivated by reuptake into the presynaptic neuron. This could mean that in some schizophrenia patients, a fast or slow COMT gene variant could potentially have the same effect as dysregulated NET expression, leading to low or high levels of extracellular NE.

Catechol-*O*-methyl-transferase is a gene that has been subject to much investigation by schizophrenia researchers (Matsuzaka et al., 2017). The COMT gene is located in a region of the 22nd chromosome of the human genome. This chromosome has been shown to harbor schizophrenia genes in genetic linkage studies. Therefore a slow or fast COMT variant may affect norepinephrine levels or at least exacerbate a problem caused by other dysregulated genes.

### Presynaptic Insulin Receptors

Phosphorylation of Akt at Ser473 can be controlled by presynaptic insulin receptors, and these insulin receptors have been shown to control NET expression (Robertson et al., 2010). Although in many schizophrenia patients, genetic dysregulation of Akt, NET, α2-adrenergic receptors or other genes could be affecting NET expression, the fact that insulin receptors can control NET via Akt phosphorylation opens up the possibility that NET dysregulation in some schizophrenia patients could be caused by aberrant insulin levels or dysfunction of the insulin receptor, leading to hyposensitivity of the insulin receptor.

As insulin resistance is seen as a hallmark of schizophrenia, it is an interesting possibility that insulin levels or lack of responsiveness to insulin levels could be affecting the function of the sympathetic nervous system and contributing to symptoms in schizophrenia patients.

### Genetic Dysregulation of β2 Adrenergic Receptor Desensitization

In some schizophrenia patients it’s possible that regulation of NET is unaffected and that it is actually the postsynaptic β2ARs that are dysregulated. Upregulation of the L-type calcium channel Cav1.2 by β2ARs is a central mechanism regulating calcium (Ca^2+^) influx into neurons.

Cav1.2 is encoded by the CACNA1C (Ca^2+^ Voltage-Gated Channel Subunit Alpha1 C) gene which has been found to be a risk gene for schizophrenia, bipolar and major depressive disorder (Bigos et al., 2010). Protein Kinase A (PKA) phosphorylation of the Cav1.2 residue S1928 displaces the β2AR from Cav1.2 upon β2AR stimulation rendering Cav1.2 refractory for several minutes from further β2AR stimulation (Patriarchi et al., 2016). This desensitization process is an important mechanism to prevent cellular over-excitation. However, if this process were dysregulated, then not only could this affect sympathetic nervous system signaling but possibly also immune cell β2AR regulation as β2ARs are present on many immune cells such as t-cells, dendritic cells and microglia.

Protein phosphatase 2 (PP2A) is required for reversal of PKA-mediated Cav1.2 channel phosphorylation. This means that PKA as well as PP2A are important for Cav1.2 regulation by phosphorylation and dephosphorylation of Ser1928 (Hall et al., 2006). As PP2A dephosphorylation of Ser1928 would result in reversing the displacement of Cav1.2 from β2ARs, a dysregulation of PP2A could lead to the inability of β2ARs to resensitize.

PP2A has been found to form a complex with β-arrestin-2 and Akt (Beaulieu et al., 2005). Research has shown that D2 receptor activation can form a β-arrestin-2/Akt/PP2A complex and that lithium disrupts the β-arrestin-2/Akt/PP2A complex by directly inhibiting GSK3 (Beaulieu et al., 2011; O’Brien et al., 2011). Although most of the research on this complex has focused on dopaminergic signaling in the striatum, it’s possible that this complex could be present in adrenergic neurons and affect β2AR desensitization, as it’s known that β-arrestin-2 is involved in the β2AR desensitization mechanism (Krasel et al., 2005).

The role of GSK3 has been discussed so far primarily in the role it could be playing in the regulation of immune tolerance within immune cells, however, it’s phosphorylation in neurons could also influence the sensitivity of β2ARs via regulation of the β-arrestin-2/Akt/PP2A complex, and could therefore be affecting signaling in the sympathetic nervous system. Research has indicated an involvement of the neuronal PI3K/Akt pathway in psychiatric disorders (Freyberg et al., 2010; Matsuda et al., 2019; Stertz et al., 2021).

GSK3β is a serine/threonine kinase that phosphorylates cellular substrates, regulating a variety of cellular functions including metabolism, protein translation, gene transcription and apoptosis, amongst other functions. The activity of GSK3β is negatively regulated by Akt and by the Wnt signaling pathway (a pathway that has been linked to schizophrenia). Most of the dysregulated pathways and genes found in schizophrenia research can be linked to GSK3β (Lovestone et al., 2007; Cole, 2013). Research has shown that antipsychotics increase the phosphorylation of GSK3, which would reduce the amount of active GSK3 (Li et al., 2007).

An interesting observation is that circadian rhythm genes CLOCK and BMAL1 can regulate the function and expression of CACNA1C in cardiac L-type calcium channels (Chen et al., 2016). If this regulation is also present in adrenergic neurons of the prefrontal cortex and sympathetic nervous system, this could result in there being a circadian control by CLOCK-BMAL1 genes of β2AR sensitivity. Although different sensitivity of β2ARs has previously been reported at different times throughout the day, most would assume that these changes in sensitivity were in response to changing extracellular NE levels. However it’s possible that CLOCK-BMAL1 genes could also be regulating β2AR sensitivity via Cav1.2 channel phosphorylation, effectively controlling the circadian sensitivity of β2ARs. As β2ARs are present in the pineal gland and the pineal gland is innervated by sympathetic nerves, a dysregulation of circadian control of β2AR sensitivity could disrupt the circadian rhythm resulting in the disturbed sleep/wake cycle seen in schizophrenia patients.

As GSK3 phosphorylation has a circadian rhythm, there may be a connection between Cav1.2 phosphorylation, CLOCK-BMAL1 genes, and GSK3 phosphorylation in relation to β2AR sensitivity, however, further research would be needed to gain a deeper understanding of a possible connection.

**FIGURE 5:**
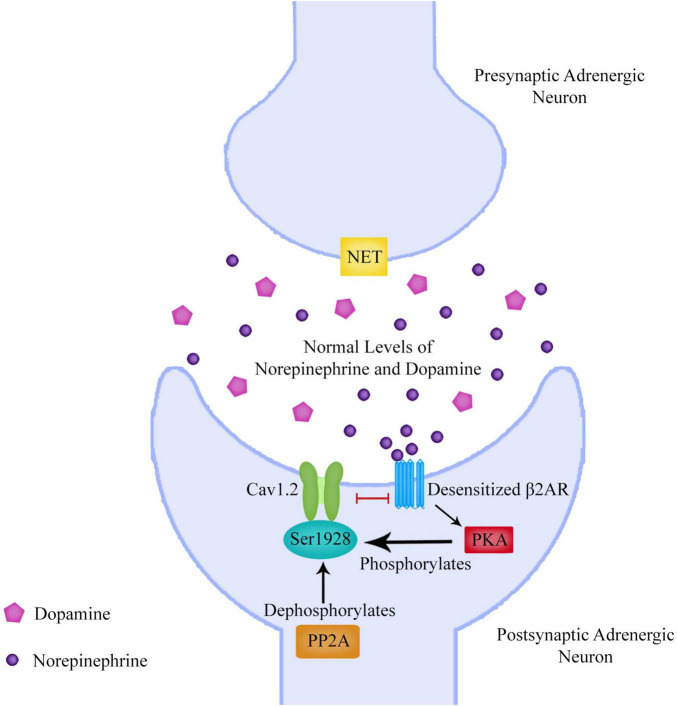
The beta-2 adrenergic receptor (β2AR) can upregulate the L-type calcium channel Cav1.2 to allow calcium entry into the neuron. Protein Kinase A (PKA) phosphorylation of the Cav1.2 residue S1928 displaces the β2AR from Cav1.2 upon β2AR stimulation rendering Cav1.2 refractory for several minutes from further β2AR stimulation. This desensitization process is an important mechanism to prevent cellular over-excitation. Protein phosphatase 2 (PP2A) is required for reversal of PKA-mediated Cav1.2 channel phosphorylation. This means that PKA as well as PP2A are important for Cav1.2 regulation by phosphorylation and dephosphorylation of Ser1928. As PP2A dephosphorylation of Ser1928 would result in reversing the displacement of Cav1.2 from β2ARs, a dysregulation of PP2A could lead to the inability of β2ARs to resensitize.

There is a possibility that in some schizophrenia patients, genetic dysregulation impairs the ability of β2ARs to adjust sensitivity in response to NE levels (Figure 5). There are some indications that dysregulation of β2AR desensitization mechanisms could also be linked to the pathophysiology of bipolar disorder (a disorder with extensive genetic overlap with schizophrenia). CACNA1C is also a risk gene for bipolar disorder and circadian rhythm genes have been found to be related to bipolar disorder risk (Shi et al., 2008; Starnawska et al., 2016).

### Intestinal Barrier and Blood Brain Barrier Permeability

In recent years there has been increasing interest in the link between the gut and the brain in schizophrenia research (Szeligowski et al., 2020). There is mounting evidence pointing toward intestinal barrier dysfunction in patients with conditions such as schizophrenia, bipolar, depression, anxiety and chronic fatigue (Severance et al., 2016; Rudzki and Szulc, 2018).

A meta-analysis carried out by the Department of Psychiatry at the University of Oxford investigated the levels of biomarkers of gut dysbiosis in patients with mental illness compared to healthy controls. Thirty three studies were included in the review, showing that patients have higher levels of zonulin, LPS, antibodies against endotoxin, sCD14 and LBP (Safadi et al., 2021). This indicates that there could be damage to the tight junctions of the intestinal epithelial barrier that is resulting in increased intestinal barrier permeability.

TNF-α levels have been found to be increased in schizophrenia patients. If schizophrenia patients are not developing the normal immune tolerance they should be in response to stress, a period of long-term stress could result in a prolonged release of pro-inflammatory cytokines such as TNF-α. Many studies have shown that cytokines, particularly TNF-α, can increase permeability of the intestinal epithelial barrier (Watson and Hughes, 2012; Al-Sadi et al., 2013; Xu et al., 2019). Transmembrane proteins in the occludin and claudin families are the main transmembrane structural elements of the tight junctions of the intestinal epithelial barrier and TNF-α has been shown to affect the expression of these proteins (Clark et al., 2015; Ni et al., 2017). The blood brain barrier (BBB) is also composed of tight junctions, and TNF-α has been found to affect BBB permeability by modulating occludin and claudin expression (Cheng et al., 2018).

**FIGURE 6:**
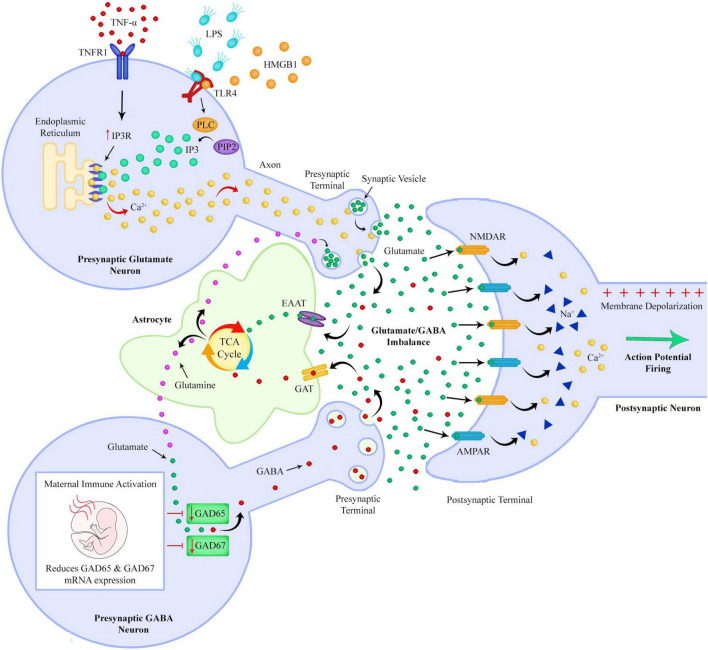
After passing the blood brain barrier, TNF-α can bind to TNF receptors on neurons resulting in an increased expression of IP3 receptors on the endoplasmic reticulum. LPS binds to TLR4s on neurons and can increase levels of IP3 by increasing PLC mediated cleavage of PIP2 to DAG and IP3. The combination of increased IP3 and IP3 receptors could lead to an excessive release of calcium from the endoplasmic reticulum, with a subsequent large increase in glutamate release into the extracellular space. If GABA synthesis is reduced due to prenatal maternal immune activation reducing expression of GAD, there could be a resultant imbalance of extracellular GABA and glutamate levels. A GABA/glutamate imbalance could lead to unsynchronized and hyperexcitable neuronal firing.

This increased intestinal barrier permeability could allow bacterial endotoxins such as LPS to pass into the circulatory system from the gut (Severance et al., 2013). LPS could then bind to TLR4s on immune cells to exacerbate production of pro-inflammatory cytokines which would further increase the amount of circulating TNF-α. LPS and pro-inflammatory cytokines such as TNF-α could pass through the compromised BBB, with LPS binding to TLR4 receptors on microglia and neurons in the brain (Figure 6). Microglial TLR4 activation could polarize microglia to an inflammatory phenotype, leading to further production of pro-inflammatory cytokines such as TNF-α (Lively and Schlichter, 2018).

### Pro-inflammatory Activation of Microglia

Microglia are the primary immune cells of the CNS. Microglia are a type of macrophage resident in the brain which interact with neurons and neuronal cells. They migrate to a source of injury or infection to remove pathogens and damaged cells.

Abnormal activation of microglia has been observed in the brains of schizophrenia patients (van Berckel et al., 2008). A recent study has shown that schizophrenia patient-derived microglia have an enhanced response to LPS, with an increased secretion of TNF-α (Ormel et al., 2020). Other studies have found that inhibition of the PI3K/Akt pathway enhances LPS-induced inflammation and increases TNF-α release (Luyendyk et al., 2008; Li M. et al., 2019).

When microglial activation was first observed in schizophrenia patients, it was generally assumed that this activation was intrinsic to the CNS. More recent research has shown that peripheral blood serum from first-episode drug-naive schizophrenia patients can provoke microglial pro-inflammatory activation (Takahashi et al., 2016; van Rees et al., 2018). This gives plausibility to the theory that the microglial activation seen in schizophrenia patients could be caused by LPS and TNF-α traveling via the circulatory system. However, it is also possible that microglia could be activated by local CNS stress-induced HMGB1 and TNF-α release, and may only be exacerbated by LPS and cytokines crossing the BBB.

Microglia can be polarized toward a pro-inflammatory or an anti-inflammatory phenotype. Microglia are polarized to an anti-inflammatory phenotype by NE acting on β2ARs, and so low sympathetic NE release or down-regulated microglial β2AR expression could result in microglia remaining in a pro-inflammatory phenotype, producing TNF-α and other harmful cytokines in response to LPS and HMGB1 (Sharma et al., 2019). This pro-inflammatory microglial activation could be the cause of the reduced gray matter volumes seen in the brain scans of schizophrenia patients (Vita et al., 2012).

### Increased Glutamate Release Due to Tumor Necrosis Factor Alpha and Lipopolysaccharide

Although it may be possible that chronic local CNS production of HMGB1 and TNF-α could be activating TLR4s on microglia and neurons, it is also possible that this activation could be exacerbated by LPS translocating from the gut. If the integrity of the BBB and intestinal barrier have both been compromised, LPS and inflammatory cytokines such as TNF-α could pass through the more permeable BBB and take effect upon neurons (Zhao et al., 2019).

It’s been shown that TNF-α can increase extracellular glutamate levels in the brain by inducing glutamate release from microglia (Takeuchi et al., 2006). Research has also shown that chronic LPS activation of neuronal TLR4 leads to glutamate release (Giansante et al., 2020).

The inositol 1,4,5-trisphosphate receptor (IP3R) is a receptor which, when activated by inositol 1,4,5-trisphosphate (IP3) in the presynaptic terminal of a glutamate neuron, releases Ca^2+^ into the terminal from the endoplasmic reticulum (ER) (Thillaiappan et al., 2019). Upon entering the presynaptic active zone Ca^2+^ then triggers neurotransmitter release within a few hundred microseconds. Ca^2+^ binds to synaptotagmins resulting in exocytosis of glutamatergic synaptic vesicles, with a resultant release of glutamate into the synaptic cleft (Südhof, 2012).

Tumor necrosis factor alpha has been found to bind to TNF receptors on neurons and upregulate the expression of IP3R on the outer surface of the ER which could result in an increase in the amount of Ca^2+^ released from the ER into the synaptic terminal (Park et al., 2009).

Research has shown that LPS also contributes to an increase of Ca^2+^ release via IP3Rs. This process is part of the endocytosis of neuronal TLR4 once it has been activated by LPS. Phospholipase y-2 (PLCγ-2) is part of the mechanism of LPS-induced TLR4 endocytosis. IP3 is the cleavage product of phosphatidylinositol 4,5-bisphosphate (PIP2) by PLCγ-2, and binds to IP3Rs on the ER resulting in the release of Ca^2+^. The increased cytosolic Ca^2+^ is required for translocation of TLR4 from the plasma membrane to endosomes (Hoffmann et al., 2003; Chiang et al., 2012; Grabbe et al., 2020).

If schizophrenia patients do have a more permeable BBB, allowing TNF-α and LPS to pass through the BBB and act upon neurons, the combination of increased LPS-induced IP3 production and TNF-α induced IP3R upregulation, could combine to result in much higher levels of Ca^2+^ being released from the ER in neurons, resulting in a large amount of excitatory glutamate release into the synaptic cleft (Figure 6). Many studies have linked perturbed Ca^2+^ signaling to psychiatric disorders such as schizophrenia (Lidow, 2003; Vidal-Domènech et al., 2020).

The schizophrenia risk gene Disrupted-in-Schizophrenia 1 (DISC1), localizes to the outer surface of the ER and has been found to regulate ER Ca^2+^ dynamics. EXOC1, a subunit of the exocyst complex, interacts with DISC1 and affects its recruitment to IP3Rs. Research has shown that knockdown of DISC1 and EXOC1 elicits an exaggerated ER Ca^2+^ response when IP3Rs are stimulated. Research has also shown dysregulated ER Ca^2+^ responses were observed in hippocampal neurons from DISC1-deficient mutant mice (Park et al., 2015).

The combined result of LPS and TNF-α acting upon glutamate neurons could be to cause a significant increase of glutamate release. This might not cause much of a problem if Gamma-aminobutyric acid (GABA) release can be increased from GABA neurons to maintain a balance of extracellular neurotransmitters. However there is considerable evidence that there is GABAergic signaling dysfunction occurring in schizophrenia patients. Deficits in inhibitory GABAergic signaling may result in an inability to maintain a neurotransmitter balance when there is a large release of extracellular glutamate.

### The Neurodevelopmental Hypothesis of Schizophrenia

Although many genes have been connected to schizophrenia, indicating a genetic component, considerable research has suggested that environmental factors may also play a part in the pathophysiology of schizophrenia (Rapoport et al., 2005). The neurodevelopmental hypothesis of schizophrenia was first proposed by Daniel Weinberger in 1987 and also discussed by R. M. Murray and S.W. Lewis in 1987 and considerable research carried out in the last few decades has bolstered this neurodevelopmental theory with increasing evidence of neurodevelopmental abnormalities contributing to the pathophysiology of schizophrenia (Murray and Lewis, 1987; Owen and O’Donovan, 2017; Weinberger, 2017). The main premise of the neurodevelopmental hypothesis is that aberrant fetal neurodevelopment creates a vulnerability to developing schizophrenia in later life.

Prenatal insults such as maternal immune activation (MIA) and prenatal iron deficiency have been linked to schizophrenia in offspring (Canetta and Brown, 2012; Aguilar-Valles et al., 2020; Choudhury and Lennox, 2021). MIA occurs when maternal inflammation during pregnancy raises inflammatory markers to above the normal range. Psychosocial stress, infection and high body mass index can all result in MIA. When the maternal immune system is triggered, cytokines are released and transmitted to the fetus, resulting in impaired neural development.

### Fetal Immune Tolerance

For a successful pregnancy, the fetus needs to maintain tolerance to both self and maternal antigens, microbes and cytokines (Rackaityte and Halkias, 2020). This raises the question as to why MIA would cause significant damage to the development of the fetus, if the fetal immune system should be suppressing itself in order to not have an excessive inflammatory response to maternal infection.

An increase in autonomic nervous system activation occurs during the transition from the 2nd into the 3rd trimester of fetal development (Cheng et al., 2004; Schneider et al., 2018). The cholinergic anti-inflammatory pathway has been found to be important in suppressing an over-inflammatory response in the fetal intestinal immune system (Garzoni et al., 2013). Immune cells are present in the fetus, with human fetuses producing dendritic cells by week 13 of pregnancy and T cells and NK cells from as early as 9 weeks. Dendritic cells are of particular importance as they are important in controlling the fetal immune response.

Dendritic cells are activated in response to PAMPs and DAMPs and possess the capacity to direct fetal T cell activation and differentiation. Research has shown that there is a large population of Treg cells in fetal lymphoid organs and blood, which play an important role in immune suppression (McGovern et al., 2017). Fetal dendritic cells migrate to lymph nodes and respond to TLR binding by promoting Treg cell induction and preventing T-cell TNF-α production via arginase-2 activity. The research conducted on this topic gives an indication that fetal tolerance depends, at least in part, on dendritic cell activation of Treg cells.

### Norepinephrine Control of Fetal Dendritic Cells

So far, the effect of reduced sympathetic NE release on ACh release from ChAT+ T cells has been discussed. However dendritic cells that are present in the intestine, the skin and lymphoid organs express adrenergic receptors and can be reached by NE released by sympathetic fibers innervating these tissues (Takenaka et al., 2016).

Research has found that NE acts upon βARs on human cord dendritic cells to inhibit the LPS stimulated production of IL-23, TNF-α and IL-6, which are cytokines that have been found to be increased in schizophrenia patients (Goyarts et al., 2008; Jang et al., 2009).

IL-23 has also been found to regulate the function of decidual immune cells during early pregnancy, with an increase of IL-23 leading to a reduction of Treg cells (Cai and Li, 2015). Tregs are a subpopulation of T cells that play a role in suppressing immune responses, thereby maintaining self-tolerance. It has been shown that Tregs are able to inhibit T cell proliferation and cytokine production (Schmidt et al., 2012).

It is possible that the mechanism by which the fetus suppresses it’s own immune system to maintain fetal tolerance, is by sympathetic neuronal NE release inhibiting IL-23 production from fetal dendritic cells. If genetic dysregulation results in a reduction in sympathetic signaling and reduced NE release in the fetus during prenatal development, levels of IL-23 could increase and this could reduce the levels of Treg cells and polarize immune cells to a more inflammatory phenotype.

This could lead to the fetus having an excessive pro-inflammatory immune response to MIA, when normally the fetal immune system would be suppressed. There is relatively little research on the mechanisms of fetal tolerance, with most research in this area focusing on the maternal immune system. However a failure of fetal immune tolerance would be a plausible explanation for the abnormal prenatal neurodevelopment widely considered to be part of the pathophysiology of schizophrenia, and so further research into fetal tolerance mechanisms could be worthwhile.

### Cholinergic Anti-inflammatory Pathway

Increased levels of vagal activity are associated with lower levels of pro-inflammatory cytokines, an effect which is mediated by the α7 nAChR on macrophages. This pathway is referred to as the cholinergic anti-inflammatory pathway and has been found to be critical in the fetus to maintain the fetal intestinal immune system. Dysregulation of this pathway has been linked to the development of necrotizing enterocolitis in the fetus (Garzoni et al., 2013).

Research has discovered that α7 nAChR stimulation by nicotine suppresses decidual M1 macrophage polarization by LPS in a preeclampsia-like mouse model (Han et al., 2021). Preeclampsia in mothers has been linked to an increased risk of schizophrenia in offspring (Lahti-Pulkkinen et al., 2020).

It is not implausible that if vagal activity is related to suppression of the immune response in the fetal intestinal immune system, that dysregulation of this pathway may also result in comprised fetal tolerance to maternal antigens, cytokines and microbes, and an increased vulnerability to MIA.

### The Prenatal Cytokine Hypothesis of Schizophrenia

Over 20 years ago Gilmore and Jarskog first proposed the hypothesis that the induction of pro-inflammatory cytokines by the maternal immune system could lead to abnormal early brain development and increase the risk of schizophrenia in offspring (Gilmore and Jarskog, 1997). High levels of cytokines in the developing fetus could affect neurodevelopment because cytokines bind to receptors on target cells, resulting in signal transduction pathways that alter gene expression in those target cells. Research has shown that after prenatal exposure to LPS, mice offspring display deficits found in schizophrenia patients including impairments in latent inhibition, prepulse inhibition, and an increased sensitivity to treatment with amphetamine (Chamera et al., 2020).

Prenatal cytokine exposure has been linked to some of the neuroanatomical abnormalities found in schizophrenia patients, including disruption of prefrontal cortical and hippocampal GABAergic markers, as well as impaired postnatal neurogenesis (Meyer et al., 2009). A loss of fetal tolerance to MIA could lead to an increase in prenatal exposure to cytokines, and this could result in the neurodevelopmental abnormalities that occur in schizophrenia patients.

### Gamma-Aminobutyric Acid Deficits in Schizophrenia

Many studies have shown alterations in GABAergic signaling in schizophrenia patients, with indications that schizophrenia patients have reduced GABA levels in the prefrontal cortex (Taylor and Tso, 2015). David W. Volk and David A. Lewis published a detailed review on this topic, considering the evidence that the cortical GABA neuron abnormalities in schizophrenia patients may arise during prenatal development (Volk and Lewis, 2014).

GABA is an inhibitory neurotransmitter. Having the correct balance of GABA and the excitatory neurotransmitter glutamate is seen as crucial to maintaining balanced neural activity. Studies which have looked at the effects of MIA on the rat fetal brain have observed alterations in GABAergic signaling (Nguyen et al., 2014; Canetta et al., 2016).

It’s been found that MIA alters GABA-synthesizing enzyme glutamic acid decarboxylase-67 (GAD67) expression in the brains of adult rat offspring (Cassella et al., 2016). It has been shown that MIA increases prefrontal levels of cytosines in the promoter regions of GAD1 and GAD2 which encode the GAD67 and GAD65 isoforms, leading to a reduction of GAD67 and GAD65 mRNA expression (Labouesse et al., 2015).

GAD67 mRNA expression has been found to be altered in schizophrenia patients (Hashimoto et al., 2003). GAD is an enzyme that catalyses the decarboxylation of glutamate to GABA and CO2. A reduction in the expression level of GAD65 and GAD67 could reduce the rate at which glutamate can be converted to GABA. The enzymes GAD65 and GAD67 maintain the main supply of GABA in the brain (de Jonge et al., 2017).

It is possible that MIA could result in reduced GABA synthesis which could lead to an imbalance of excitatory and inhibitory neurotransmitters, with reduced levels of GABA released into the synaptic cleft during neurotransmission.

### The Effect of a Glutamate/Gamma-Aminobutyric Acid Imbalance on Striatal Dopamine Signaling

The dopamine hypothesis of schizophrenia has been a focus of much research for many decades. The idea that dysregulated DA levels contribute to the pathophysiology of schizophrenia was an idea that was formed by research in the 1960s and 1970s which showed that amphetamine drugs which increase DA could cause psychosis, and DA receptor blockers could alleviate psychosis symptoms. This hypothesis has evolved and refined over time and increasing evidence points to there being low DA levels in the prefrontal cortex and high levels of DA in the striatum of schizophrenia patients (Brisch et al., 2014).

Although the theory in this paper gives a clear explanation of how upregulated NET expression could lead to lower levels of DA in the prefrontal cortex, there is still the unanswered question of why DA levels would be increased in the striatum.

As mentioned earlier, research has pointed toward an imbalance in the neurotransmitters glutamate and GABA as a cause of the positive symptoms of schizophrenia (Koshiyama et al., 2018; Bojesen et al., 2020). If, as this paper has suggested, there is an excessive release of glutamate without an increase in inhibitory GABA release, this could lead to glutamate excitotoxicity. Research has found indications of glutamate excitotoxicity in schizophrenia patients (Li C. T. et al., 2019).

It is possible that a glutamate/GABA imbalance could also lead to dysregulated DA signaling in the striatum. It has been found that GABA-A receptors control striatal DA release (Kramer et al., 2020). The reduced ability to synthesize GABA due to MIA-related aberrant neurodevelopment could therefore lead to a lack of inhibition of DA neurons and an increased release of DA in the striatum (Creed et al., 2014; McCutcheon et al., 2019).

Research has shown that striatal-projecting DA neurons are involved in the avoidance of threatening stimuli (Menegas et al., 2018). If DA is dysregulated in the striatum of schizophrenia patients, their ability to correctly perceive what is and isn’t a threat could be compromised. This may explain the paranoia and feelings of persecution that are among the positive symptoms of schizophrenia.

As well as there being a lack of inhibitory GABA signaling, it’s also possible that the striatal DAT is dysregulated. It has already been described in this paper, how dysregulated insulin receptor-Akt-NET signaling could lead to increased NET membrane expression in the prefrontal cortex, and result in increased DA uptake and increased NE content in presynaptic vesicles. Considering that the striatal DAT is also regulated by an insulin-Akt pathway, it’s possible that genetic dysregulation of this pathway could also affect DA uptake in the striatum (Speed et al., 2011). Although there has been some research showing dysregulated expression of DAT in the striatum of schizophrenia patients, further research is needed to understand these mechanisms more clearly (Sekiguchi et al., 2019).

## Discussion

### Different Types of Schizophrenia

The theory of schizophrenia suggested in this paper describes an immune dysregulation type of schizophrenia. However, it is possible, and quite likely, that there could be different types of schizophrenia. It may be possible to broadly split schizophrenia into immune activation and non-immune activation types of schizophrenia.

A study carried out by researchers at the University of Pennsylvania observed brain scans of schizophrenia patients and found that approximately 60% of patients had signs of inflammation and decreased gray matter, while the remaining 40% showed no such changes (Chand et al., 2020). It’s possible that the reduced gray matter could be due to microglial pro-inflammatory activation. It could be that the 60% of patients with brains showing reduced gray matter had immune activation schizophrenia, and the remaining 40% of patients may have had non-immune activation schizophrenia.

### Consideration of Existing Hypotheses

The hypothesis proposed in this paper actually combines multiple existing hypotheses into a quite specific hypothetical model of the pathophysiology of schizophrenia. This model encompasses well-established theories such as the two-hit model of schizophrenia, the neurodevelopmental hypothesis, the reduced GABA hypothesis and also the dopamine hypothesis which in recent years has suggested hypodopaminergic signaling in the prefrontal cortex and hyperdopaminergic signaling in the striatum of schizophrenia patients (Murray and Lewis, 1987; Petty, 1995; Bayer et al., 1999; Feigenson et al., 2014; Weinberger, 2017).

Also considered here are more recent ideas such as the possibility of endotoxin translocation in schizophrenia patients, a concept that has been considered more broadly in the endotoxin hypothesis of neurodegeneration, a hypothesis which, despite focusing mostly on the role of endotoxin in Alzheimer’s disease, does suggest the possibility of endotoxin activation of microglia in schizophrenia patients (Brown, 2019).

The model proposed in this paper also aligns with previous immune hypotheses which have suggested that prenatal adversity affects immune system development, and also aligns with the microglia hypothesis of schizophrenia which suggests excessive microglial activation and pro-inflammatory cytokine release is involved in schizophrenia pathophysiology (Monji et al., 2009; Kinney et al., 2010).

An autonomic hypothesis of schizophrenia has been proposed before, suggesting that a dysregulation of vagus nerve control of immune cells may contribute to schizophrenia pathophysiology (das Graças Corsi-Zuelli et al., 2017; Stogios et al., 2021).

The muscarinic hypothesis of schizophrenia implies a role of muscarinic receptors in schizophrenia with increasing evidence suggesting that targeting muscarinic receptor subtypes can modulate the specific brain circuits that are disrupted in schizophrenia (Raedler et al., 2006; Foster et al., 2021). ACh has been found to excite sympathetic neurons via M1 muscarinic receptors, which indicates that dysfunction of these receptors could affect sympathetic nervous system function and consequent immune cell dysregulation.

What seems to be a novel idea in this paper is the suggestion that autonomic nervous system dysregulation in the fetus affects control of the mechanisms of fetal immune tolerance which would normally suppress the fetal immune system from excess pro-inflammatory activation in response to MIA.

Previous research has suggested that increased cortical NET expression could lead to excessive presynaptic uptake of NE and DA, increased DA to NE conversion, and consequent high presynaptic levels of NE and reduced levels of DA in the cortex. Although this is not a new idea, what does appear to be a novel idea is suggesting that this could be leading to sympathetic nervous system dysregulation and alterations in immune cell activation.

Another novel concept suggested in this paper is that β2AR desensitization, either by excessive stress-induced release of NE or genetic dysregulation of β2AR sensitization mechanisms, dysregulates the sympathetic nervous system leading to altered immune cell function in schizophrenia patients.

The possibility that stress-induced HMGB1 release could chronically activate the immune system in schizophrenia patients due to dysfunction of immune tolerance mechanisms is another novel concept put forward in this paper.

This paper aims to tie together many pre-existing hypotheses with a number of new concepts to propose a convincing hypothetical model of schizophrenia.

### Limitations in Interpreting Data

Trying to find consistent patterns of immune activation in schizophrenia patients is made somewhat difficult by the fact that many studies are conducted on patients taking antipsychotic medication, which could either correct, overcorrect or dysregulate immune cell function. There is considerable evidence that antipsychotics dysregulate the immune system, with evidence that certain antipsychotics are immunosuppressive and can reduce pro-inflammatory cytokine production in response to LPS (Romeo et al., 2018; May et al., 2019).

The interpretation of research findings is also complicated by the different receptor targets and mechanisms of action of different types of antipsychotics, and the fact that each schizophrenia patient may have different mechanisms of immune dysregulation, or none at all depending on their personal combination of dysregulated genes and/or specific environmental factors.

An important challenge faced by schizophrenia researchers is to aim to understand whether the suppression of the immune response by antipsychotics is part of their mechanism of action to correct dysregulation and alleviate symptoms in patients, or whether it is just a side effect of antipsychotic drugs.

Indeed, even if the immune system is consistently shown to be polarized to a pro-inflammatory state in antipsychotic-naive patients, how can we know if this is actually a core part of the pathophysiology of the disorder, rather than being more of a side story which, despite possibly causing some minor symptoms, is not responsible for the main positive and negative symptoms seen in schizophrenia patients.

Although studies testing levels of cytokines, Treg cells, and immune cell responses to activation are very useful as a way of detecting patterns of immune activation, they will not necessarily help us to understand the reasons for that activation and what part that activation plays in schizophrenia.

A more constructive approach might be to use the general patterns emerging from the data on immune activation in schizophrenia patients to generate hypothetical models of the most logical and likely pathway that could be causing this dysregulation, and then carry out research testing these hypothetical models until they are generally proven to be correct or incorrect. This process of logic and elimination may edge researchers closer to understanding not just whether there is immune activation in schizophrenia patients but whether it actually plays a relevant part in the pathophysiology of schizophrenia, and what part that is.

Perceiving schizophrenia patients as one homogeneous group when interpreting data may make it difficult to find consistent correlations in data. For example, if in a particular study 10% of patients show increased immune activity and 90% show no immune dysfunction, rather than perceiving schizophrenia patients as mostly having unaltered immune responses, the next logical step would be to work out if the 10% have a specific immune dysregulation type of schizophrenia and carry out further research on these particular patients, for example to see if there is a higher frequency of certain gene variants or a particularly distinct symptomatology in the immune dysfunction group.

This approach is more likely to be effective if it is the case that schizophrenia patients are actually a group of people who, despite having similar symptoms, actually have different biological dysregulation causing those symptoms. A good idea might be to genetically test participants in immune research studies, to work out if specific immune signatures correlate with specific genetic variants.

## Conclusion

Although many patients respond to antipsychotic medication, there are a significant number of patients who do not have an adequate response to medication. Both typical and a-typical medications can cause unwanted side effects such as weight gain and extrapyramidal side effects.

Having a better understanding of the dysregulated biological pathways that cause schizophrenia opens up some interesting possibilities in terms of improving the patient’s treatment experience. For example, if certain genetic variants are connected to a specific biological pathway, this opens up the possibility of using gene testing to predict the antipsychotic drug that might be most suited to a patient. This could result in the patient spending less time in hospital, and being less likely to discontinue treatment due to unpleasant side effects.

Many schizophrenia patients suffer from paranoia and a lack of insight. This can make it difficult to persuade them to seek medical treatment when they are suffering from psychosis. This means that patients can spend years suffering from psychosis before receiving treatment. This can have a drastic and damaging effect on their relationships, career, finances and physical health, as well as being stressful for their family members.

If there is an immune dysregulation type of schizophrenia, it may be possible to identify biomarkers by testing for an illness-specific immune signature in blood serum. Screening could then be carried out to identify those people who are at risk of developing the disease and preventative treatment could be administered to prevent psychosis from developing. It is standard practice to screen for certain types of cancer, and so screening and prevention for mental illnesses may be a possibility in the future.

## Data Availability Statement

The original contributions presented in this study are included in the article/supplementary material, further inquiries can be directed to the corresponding author.

## Author Contributions

TC confirmed sole responsibility for the following: theory/hypothesis conception, research, analysis and interpretation of research, and manuscript preparation.

## Conflict of Interest

The author declares that the research was conducted in the absence of any commercial or financial relationships that could be construed as a potential conflict of interest.

## Publisher’s Note

All claims expressed in this article are solely those of the authors and do not necessarily represent those of their affiliated organizations, or those of the publisher, the editors and the reviewers. Any product that may be evaluated in this article, or claim that may be made by its manufacturer, is not guaranteed or endorsed by the publisher.
